# What influences students’ abilities to critically evaluate scientific investigations?

**DOI:** 10.1371/journal.pone.0273337

**Published:** 2022-08-30

**Authors:** Ashley B. Heim, Cole Walsh, David Esparza, Michelle K. Smith, N. G. Holmes

**Affiliations:** 1 Department of Ecology and Evolutionary Biology, Cornell University, Ithaca, NY, United States of America; 2 Laboratory of Atomic and Solid State Physics, Cornell University, Ithaca, NY, United States of America; University of Belgrade Faculty of Organisational Sciences: Univerzitet u Beogradu Fakultet organizacionih nauka, SERBIA

## Abstract

Critical thinking is the process by which people make decisions about what to trust and what to do. Many undergraduate courses, such as those in biology and physics, include critical thinking as an important learning goal. Assessing critical thinking, however, is non-trivial, with mixed recommendations for how to assess critical thinking as part of instruction. Here we evaluate the efficacy of assessment questions to probe students’ critical thinking skills in the context of biology and physics. We use two research-based standardized critical thinking instruments known as the Biology Lab Inventory of Critical Thinking in Ecology (Eco-BLIC) and Physics Lab Inventory of Critical Thinking (PLIC). These instruments provide experimental scenarios and pose questions asking students to evaluate what to trust and what to do regarding the quality of experimental designs and data. Using more than 3000 student responses from over 20 institutions, we sought to understand what features of the assessment questions elicit student critical thinking. Specifically, we investigated (a) how students critically evaluate aspects of research studies in biology and physics when they are individually evaluating one study at a time versus comparing and contrasting two and (b) whether individual evaluation questions are needed to encourage students to engage in critical thinking when comparing and contrasting. We found that students are more critical when making comparisons between two studies than when evaluating each study individually. Also, compare-and-contrast questions are sufficient for eliciting critical thinking, with students providing similar answers regardless of if the individual evaluation questions are included. This research offers new insight on the types of assessment questions that elicit critical thinking at the introductory undergraduate level; specifically, we recommend instructors incorporate more compare-and-contrast questions related to experimental design in their courses and assessments.

## Introduction

### Critical thinking and its importance

Critical thinking, defined here as “the ways in which one uses data and evidence to make decisions about what to trust and what to do” [1], is a foundational learning goal for almost any undergraduate course and can be integrated in many points in the undergraduate curriculum. Beyond the classroom, critical thinking skills are important so that students are able to effectively evaluate data presented to them in a society where information is so readily accessible [2, 3]. Furthermore, critical thinking is consistently ranked as one of the most necessary outcomes of post-secondary education for career advancement by employers [4]. In the workplace, those with critical thinking skills are more competitive because employers assume they can make evidence-based decisions based on multiple perspectives, keep an open mind, and acknowledge personal limitations [5, 6]. Despite the importance of critical thinking skills, there are mixed recommendations on how to elicit and assess critical thinking during and as a result of instruction. In response, here we evaluate the degree to which different critical thinking questions elicit students’ critical thinking skills.

### Assessing critical thinking in STEM

Across STEM (i.e., science, technology, engineering, and mathematics) disciplines, several standardized assessments probe critical thinking skills. These assessments focus on aspects of critical thinking and ask students to evaluate experimental methods [7–11], form hypotheses and make predictions [12, 13], evaluate data [2, 12–14], or draw conclusions based on a scenario or figure [2, 12–14]. Many of these assessments are open-response, so they can be difficult to score, and several are not freely available.

In addition, there is an ongoing debate regarding whether critical thinking is a domain-general or context-specific skill. That is, can someone transfer their critical thinking skills from one domain or context to another (domain-general) or do their critical thinking skills only apply in their domain or context of expertise (context-specific)? Research on the effectiveness of teaching critical thinking has found mixed results, primarily due to a lack of consensus definition of and assessment tools for critical thinking [15, 16]. Some argue that critical thinking is domain-general—or what Ennis refers to as the “general approach”—because it is an overlapping skill that people use in various aspects of their lives [17]. In contrast, others argue that critical thinking must be elicited in a context-specific domain, as prior knowledge is needed to make informed decisions in one’s discipline [18, 19]. Current assessments include domain-general components [2, 7, 8, 14, 20, 21], asking students to evaluate, for instance, experiments on the effectiveness of dietary supplements in athletes [20] and context-specific components, such as to measure students’ abilities to think critically in domains such as neuroscience [9] and biology [10].

Others maintain the view that critical thinking is a context-specific skill for the purpose of undergraduate education, but argue that it should be content accessible [22–24], as “thought processes are intertwined with what is being thought about” [23]. From this viewpoint, the context of the assessment would need to be embedded in a relatively accessible context to assess critical thinking independent of students’ content knowledge. Thus, to effectively elicit critical thinking among students, instructors should use assessments that present students with accessible domain-specific information needed to think deeply about the questions being asked [24, 25].

Within the context of STEM, current critical thinking assessments primarily ask students to evaluate a single experimental scenario (e.g., [10, 20]), though compare-and-contrast questions about more than one scenario can be a powerful way to elicit critical thinking [26, 27]. Generally included in the “Analysis” level of Bloom’s taxonomy [28–30], compare-and-contrast questions encourage students to recognize, distinguish between, and relate features between scenarios and discern relevant patterns or trends, rather than compile lists of important features [26]. For example, a compare-and-contrast assessment may ask students to compare the hypotheses and research methods used in two different experimental scenarios, instead of having them evaluate the research methods of a single experiment. Alternatively, students may inherently recall and use experimental scenarios based on their prior experiences and knowledge as they evaluate an individual scenario. In addition, evaluating a single experimental scenario individually may act as metacognitive scaffolding [31, 32]—a process which “guides students by asking questions about the task or suggesting relevant domain-independent strategies [32]—to support students in their compare-and-contrast thinking.

### Purpose and research questions

Our primary objective of this study was to better understand what features of assessment questions elicit student critical thinking using two existing instruments in STEM: the Biology Lab Inventory of Critical Thinking in Ecology (Eco-BLIC) and Physics Lab Inventory of Critical Thinking (PLIC). We focused on biology and physics since critical thinking assessments were already available for these disciplines. Specifically, we investigated (a) how students critically evaluate aspects of research studies in biology and physics when they are individually evaluating one study at a time or comparing and contrasting two studies and (b) whether individual evaluation questions are needed to encourage students to engage in critical thinking when comparing and contrasting.

Providing undergraduates with ample opportunities to practice critical thinking skills in the classroom is necessary for evidence-based critical thinking in their future careers and everyday life. While most critical thinking instruments in biology and physics contexts have undergone some form of validation to ensure they are accurately measuring the intended construct, to our knowledge none have explored how different question types influence students’ critical thinking. This research offers new insight on the types of questions that elicit critical thinking, which can further be applied by educators and researchers across disciplines to measure cognitive student outcomes and incorporate more effective critical thinking opportunities in the classroom.

## Methods

### Ethics statement

The procedures for this study were approved by the Institutional Review Board of Cornell University (Eco-BLIC: #1904008779; PLIC: #1608006532). Informed consent was obtained by all participating students via online consent forms at the beginning of the study, and students did not receive compensation for participating in this study unless their instructor offered credit for completing the assessment.

### Participants and assessment distribution

We administered the Eco-BLIC to undergraduate students across 26 courses at 11 institutions (six doctoral-granting, three Master’s-granting, and two Baccalaureate-granting) in Fall 2020 and Spring 2021 and received 1612 usable responses. Additionally, we administered the PLIC to undergraduate students across 21 courses at 11 institutions (six doctoral-granting, one Master’s-granting, three four-year colleges, and one 2-year college) in Fall 2020 and Spring 2021 and received 1839 usable responses. We recruited participants via convenience sampling by emailing instructors of primarily introductory ecology-focused courses or introductory physics courses who expressed potential interest in implementing our instrument in their course(s). Both instruments were administered online via Qualtrics and students were allowed to complete the assessments outside of class. The demographic distribution of the response data is presented in Table 1, all of which were self-reported by students. The values presented in this table represent all responses we received.

**Table 1:** Submitted responses broken down by demographic information.

**Gender**	**Eco-BLIC**	**PLIC**
Woman	58.3%	39.5%
Man	39.4%	51.3%
Non-binary/Non-gender conforming	1.0%	1.8%
Self-describe	0.2%	7.3%
Prefer not to disclose	1.1%	0%
**Race/Ethnicity**	**Eco-BLIC**	**PLIC**
American Indian or Alaska Native	1.3%	0.8%
Asian	16.8%	27.6%
Black or African American	5.4%	5.1%
Hispanic or Latinx	19.6%	8.8%
Native Hawaiian / Pacific Islander	0.4%	0.4%
White	53.5%	53.3%
Self-describe / Prefer not to disclose / Other	3.1%	1.7%
**Major**	**Eco-BLIC**	**PLIC**
Ecology & Evolutionary Biology	21.28%	
Molecular Biology	16.25%	
Physiology or Neuroscience	10.86%	
No specialization / I don’t know	16.07%	
Non-Life Science Major	35.55%	
Engineering		45.8%
Other science		19.0%
Physics		17.7%
Non-science		6.6%
Unknown		10.9%

### Instrument description

#### Question types

Though the content and concepts featured in the Eco-BLIC and PLIC are distinct, both instruments share a similar structure and set of question types. The Eco-BLIC—which was developed using a structure similar to that of the PLIC [1]—includes two predator-prey scenarios based on relationships between (a) smallmouth bass and mayflies and (b) great-horned owls and house mice. Within each scenario, students are presented with a field-based study and a laboratory-based study focused on a common research question about feeding behaviors of smallmouth bass or house mice, respectively. The prompts for these two Eco-BLIC scenarios are available in S1 and S2 Appendices. The PLIC focuses on two research groups conducting different experiments to test the relationship between oscillation periods of masses hanging on springs [1]; the prompts for this scenario can be found in S3 Appendix. The descriptive prompts in both the Eco-BLIC and PLIC also include a figure presenting data collected by each research group, from which students are expected to draw conclusions. The research scenarios (e.g., field-based group and lab-based group on the Eco-BLIC) are written so that each group has both strengths and weaknesses in their experimental designs.

After reading the prompt for the first experimental group (Group 1) in each instrument, students are asked to identify possible claims from Group 1’s data (data evaluation questions). Students next evaluate the strengths and weaknesses of various study features for Group 1 (individual evaluation questions). Examples of these individual evaluation questions are in Table 2. They then suggest next steps the group should pursue (next steps items). Students are then asked to read about the prompt describing the second experimental group’s study (Group 2) and again answer questions about the possible claims, strengths and weaknesses, and next steps of Group 2’s study (data evaluation questions, individual evaluation questions, and next steps items). Once students have independently evaluated Groups 1 and 2, they answer a series of questions to compare the study approaches of Group 1 versus Group 2 (group comparison items). In this study, we focus our analysis on the individual evaluation questions and group comparison items.

**Table 2:** Examples of individual evaluation and group comparison items on the Eco-BLIC and PLIC.

Type of Question	Eco-BLIC (Owl/Mouse Scenario—Lab Group)	PLIC
Individual evaluation questions Response type: *Single choice closed response (from 1-weakness to 4-strength)*	Please characterize each of the following aspects of Group 1’s study setup as either a strength or weakness to defining the feeding behavior of mice while great-horned owl calls play: *Conducting study over one night*	Please characterize the following aspects of Group 1’s data collection methods as either a strength or weakness of their methods: *The variables measured (time and mass)*
Group comparison items Response type: *Single choice closed response* *Group 1 was more effective* *Group 2 was more effective* *Both groups were highly effective* *Both groups were minimally effective*	How do you think Group 1 and Group 2 performed in the following categories? *Used an appropriate duration of time for the study (Group 1*: *one night; Group 2*: *two nights)*	How do you think Group 1 and Group 2 performed in the following categories related to data collection methods? *The variables measured (time and mass)*

The Eco-BLIC examples are derived from the owl/mouse scenario.

#### Instrument versions

To determine whether the individual evaluation questions impacted the assessment of students’ critical thinking, students were randomly assigned to take one of two versions of the assessment via Qualtrics branch logic: 1) a version that included the individual evaluation and group comparison items or 2) a version with only the group comparison items, with the individual evaluation questions removed. We calculated the median time it took students to answer each of these versions for both the Eco-BLIC and PLIC.

#### Think-aloud interviews

We also conducted one-on-one think-aloud interviews with students to elicit feedback on the assessment questions (Eco-BLIC n = 21; PLIC n = 4). Students were recruited via convenience sampling at our home institution and were primarily majoring in biology or physics. All interviews were audio-recorded and screen captured via Zoom and lasted approximately 30–60 minutes. We asked participants to discuss their reasoning for answering each question as they progressed through the instrument. We did not analyze these interviews in detail, but rather used them to extract relevant examples of critical thinking that helped to explain our quantitative findings. Multiple think-aloud interviews were conducted with students using previous versions of the PLIC [1], though these data are not discussed here.

#### Data analyses

**Fig 1:**
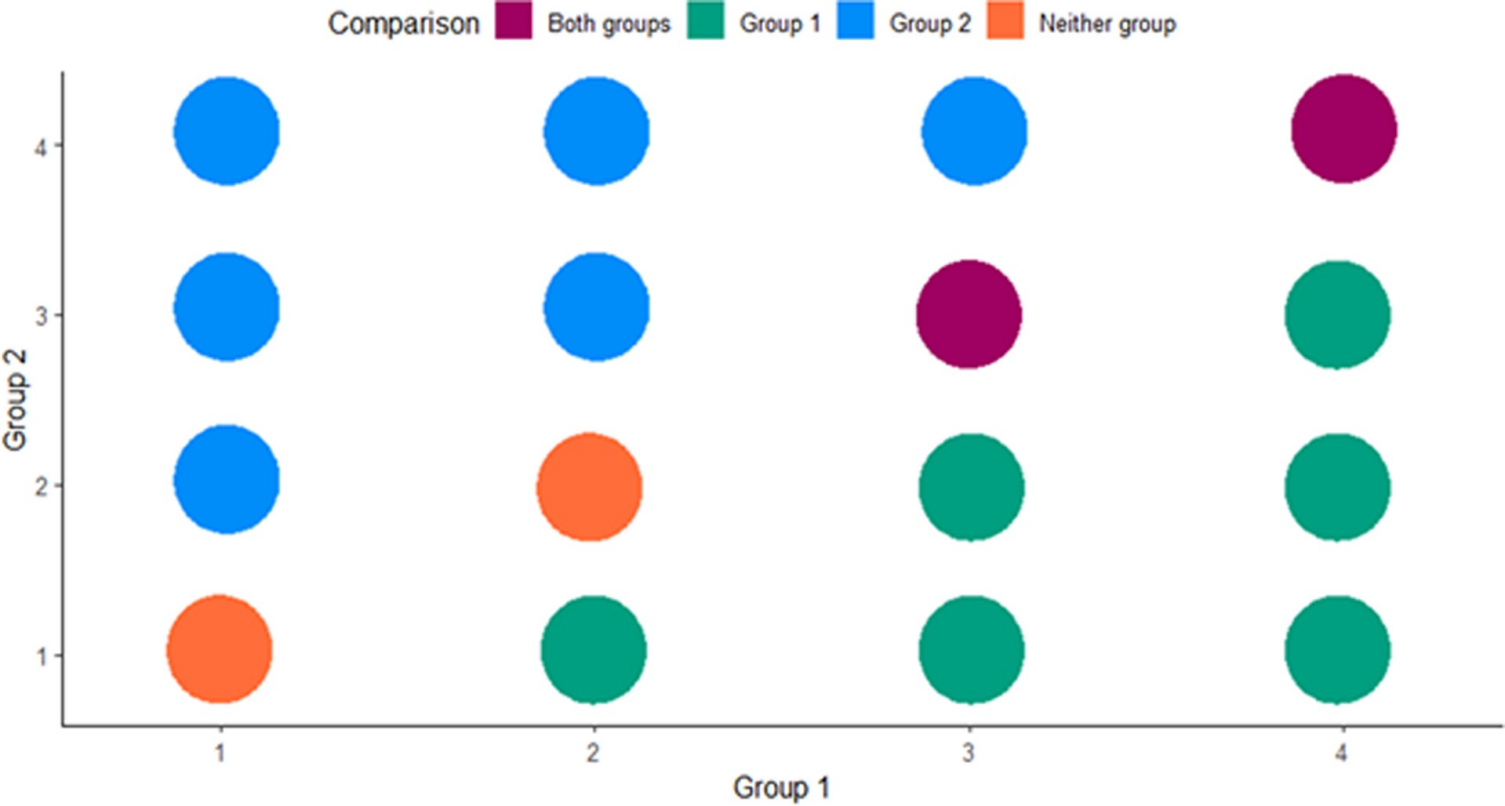
Idealized multi-pie chart graph representing expected alignment between individual evaluation and group comparison items on the Eco-BLIC and PLIC. The x- and y-axes represent rankings on the individual evaluation questions for Groups 1 and 2 (or field and lab groups), respectively. The colors in the legend at the top of the figure denote responses to the group comparison items. In this idealized example, all pie charts are the same size to indicate that the student answers are equally proportioned across all answer combinations.

Our analyses focused on (1) investigating the alignment between students’ responses to the individual evaluation questions and the group comparison items and (2) comparing student responses between the two instrument versions. If individual evaluation and group comparison items elicit critical thinking in the same way, we would expect to see the same frequency of responses for each question type, as per Fig 1. For example, if students evaluated one study feature of Group 1 as a strength and the same study feature for Group 2 as a strength, we would expect that students would respond that both groups were highly effective for this study feature on the group comparison item (i.e., data represented by the purple circle in the top right quadrant of Fig 1). Alternatively, if students evaluated one study feature of Group 1 as a strength and the same study feature for Group 2 as a weakness, we would expect that students would indicate that Group 1 was more effective than Group 2 on the group comparison item (i.e., data represented by the green circle in the lower right quadrant of Fig 1).

We ran descriptive statistics to summarize student responses to questions and examine distributions and frequencies of the data on the Eco-BLIC and PLIC. We also conducted chi-square goodness-of-fit tests to analyze differences in student responses between versions within the relevant questions from the same instrument. In all of these tests, we used a Bonferroni correction to lower the chances of receiving a false positive and account for multiple comparisons. We generated figures—primarily multi-pie chart graphs and heat maps—to visualize differences between individual evaluation and group comparison items and between versions of each instrument with and without individual evaluation questions, respectively. All aforementioned data analyses and figures were conducted or generated in the R statistical computing environment (v. 4.1.1) and Microsoft Excel.

## Results

**Fig 2:**
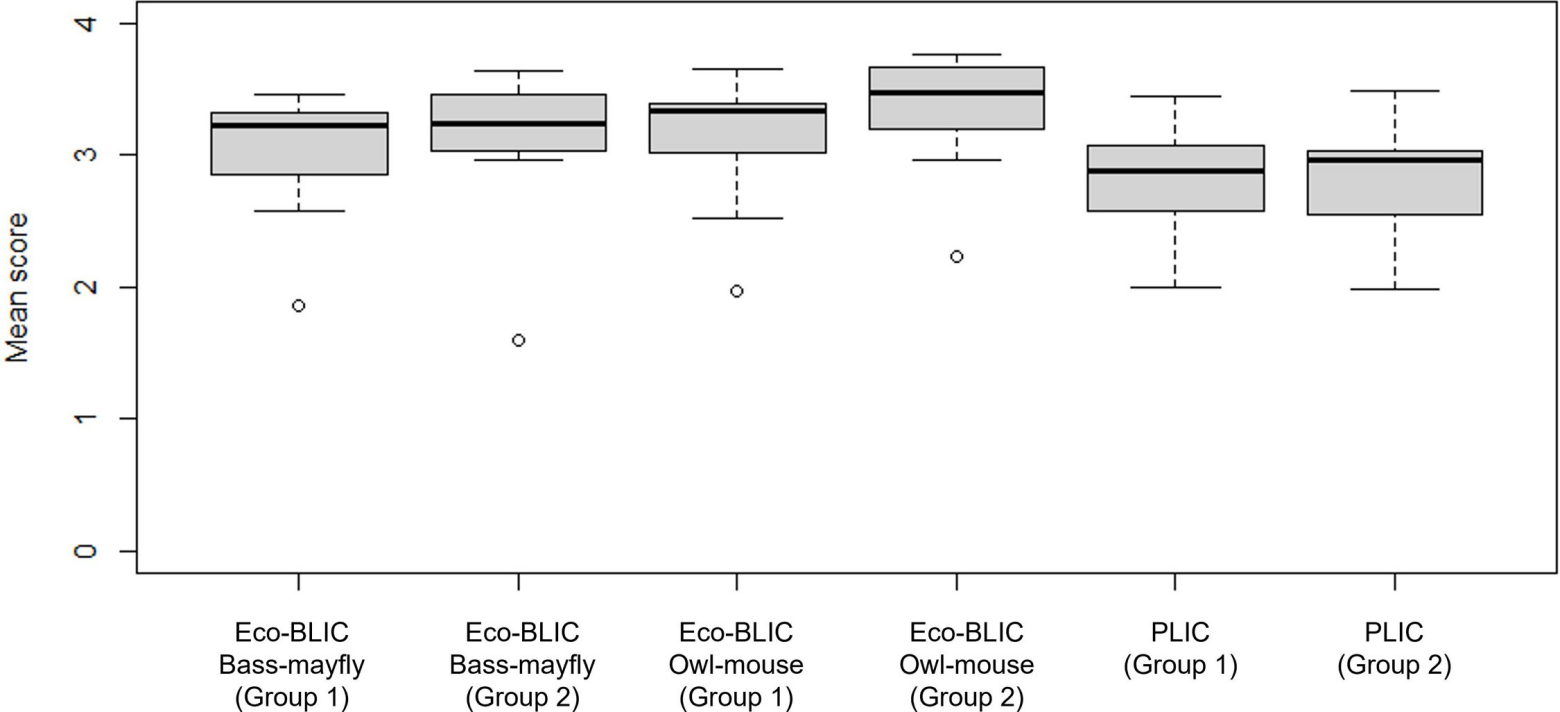
Boxplots representing mean scores and standard errors of individual evaluation questions for Group 1 and Group 2 across the Eco-BLIC and PLIC scenarios. Each box represents the interquartile range (IQR). Lines within each box represent the median. Circles represent outliers of mean scores for each question.

We asked students to evaluate different experimental set-ups on the Eco-BLIC and PLIC two ways. Students first evaluated the strengths and weaknesses of study features for each scenario individually (individual evaluation questions, Table 2) and, subsequently, answered a series of questions to compare and contrast the study approaches of both research groups side-by-side (group comparison items, Table 2). Through analyzing the individual evaluation questions, we found that students generally ranked experimental features (i.e., those related to study set-up, data collection and summary methods, and analysis and outcomes) of the independent research groups as strengths (Fig 2), evidenced by the mean scores greater than 2 on a scale from 1 (weakness) to 4 (strength).

### Individual evaluation versus compare-and-contrast evaluation

**Fig 3:**
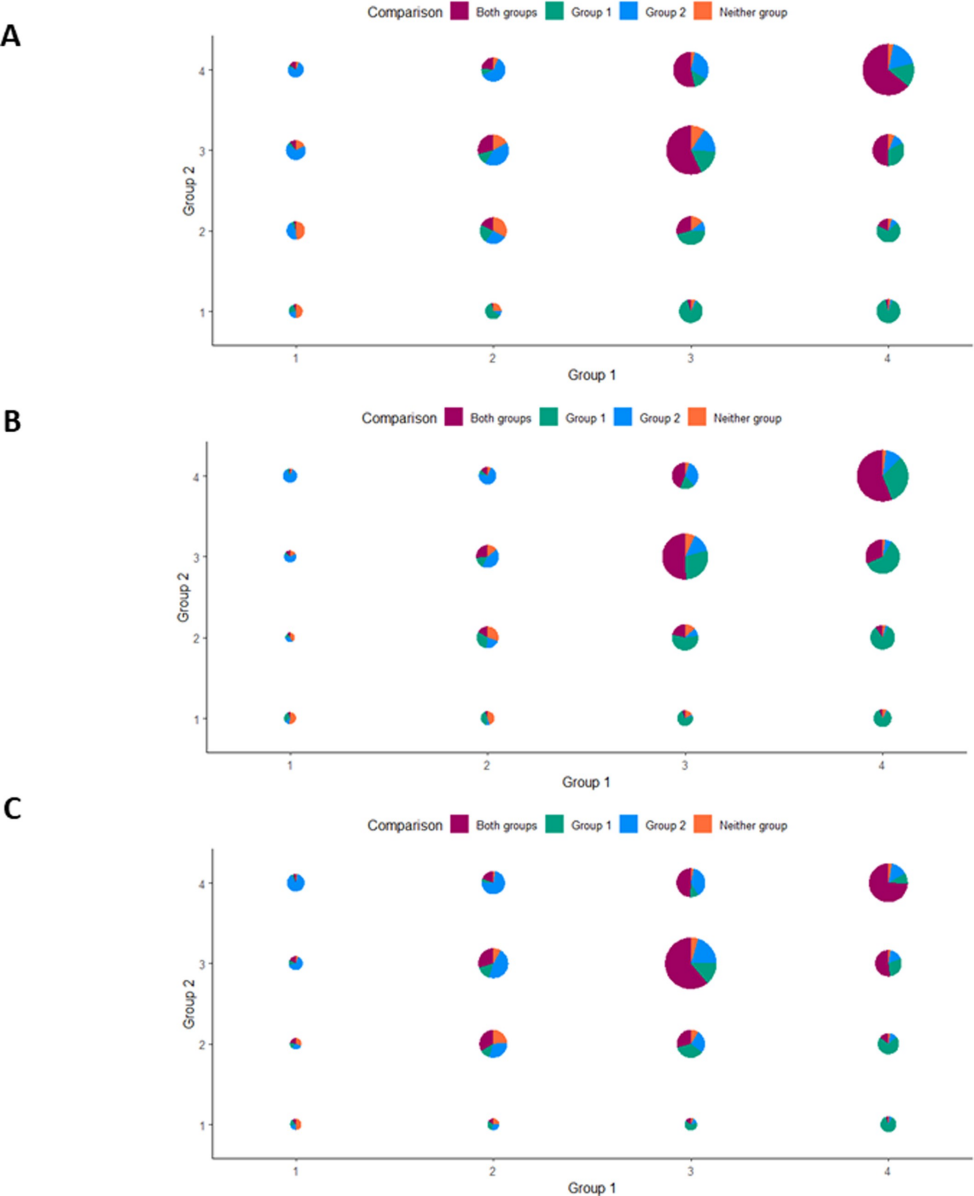
Alignment between individual evaluation and group comparison items. The x- and y-axes represent students’ rankings on the individual evaluation questions for Groups 1 and 2 on each assessment, respectively, where 1 indicates weakness and 4 indicates strength. The overall size of each pie chart represents the proportion of students who responded with each pair of ratings. The colors in the pie charts denote the proportion of students’ responses who chose each option on the group comparison items. (A) Eco-BLIC bass-mayfly scenario (B) Eco-BLIC owl-mouse scenario (C) PLIC oscillation periods of masses hanging on springs scenario.

Our results indicate that when students consider Group 1 or Group 2 individually, they mark most study features as strengths (consistent with the means in Fig 2), shown by the large circles in the upper right quadrant across the three experimental scenarios (Fig 3). However, the proportion of colors on each pie chart shows that students select a range of responses when comparing the two groups [e.g., Group 1 being more effective (green), Group 2 being more effective (blue), both groups being effective (purple), and neither group being effective (orange)]. We infer that students were more discerning (i.e., more selective) when they were asked to compare the two groups across the various study features (Fig 3). In short, students think about the groups differently if they are rating either Group 1 or Group 2 in the individual evaluation questions versus directly comparing Group 1 to Group 2.

These results are further supported by student responses from the think-aloud interviews. For example, one interview participant responding to the bass-mayfly scenario of the Eco-BLIC explained that accounting for bias/error in both the field and lab groups in this scenario was a strength (i.e., 4). This participant mentioned that Group 1, who performed the experiment in the field, “[had] outliers, so they must have done pretty well,” and that Group 2, who collected organisms in the field but studied them in lab, “did a good job of accounting for bias.” However, when asked to compare between the groups, this student argued that Group 2 was more effective at accounting for bias/error, noting that “they controlled for more variables.”

Another individual who was evaluating “repeated trials for each mass” in the PLIC expressed a similar pattern. In response to ranking this feature of Group 1 as a strength, they explained: “Given their uncertainties and how small they are, [the group] seems like they’ve covered their bases pretty well.” Similarly, they evaluated this feature of Group 2 as a strength as well, simply noting: “Same as the last [group], I think it’s a strength.” However, when asked to compare between Groups 1 and 2, this individual argued that Group 1 was more effective because they conducted more trials.

### Individual evaluation questions to support compare and contrast thinking

**Fig 4:**
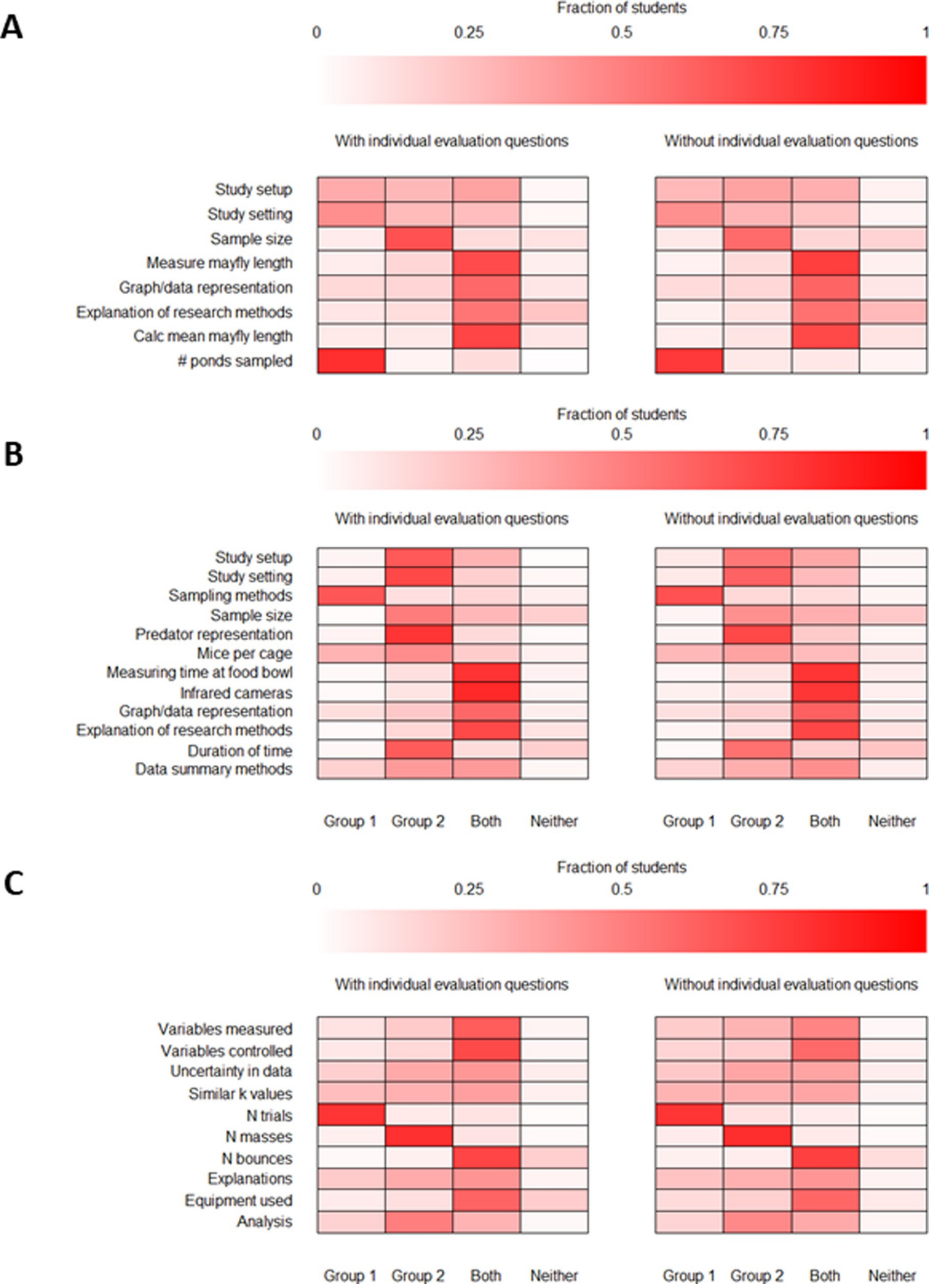
Heat maps representing differences in student responses on the group comparison items with and without individual evaluation questions. The x-axis denotes students’ responses on the group comparison items (i.e., whether they ranked Group 1 as more effective, Group 2 as more effective, both groups as highly effective, or neither group as effective/both groups were minimally effective). The y-axis lists each of the study features that students compared between the field and lab groups. White and lighter shades of red indicate a lower percentage of student responses, while brighter red indicates a higher percentage of student responses. (A) Eco-BLIC bass-mayfly scenario. (B) Eco-BLIC owl-mouse scenario. (C) PLIC oscillation periods of masses hanging on springs scenario.

Given that students were more discerning when they directly compared two groups for both biology and physics experimental scenarios, we next sought to determine if the individual evaluation questions for Group 1 or Group 2 were necessary to elicit or helpful to support student critical thinking about the investigations. To test this, students were randomly assigned to one of two versions of the instrument. Students in one version saw individual evaluation questions about Group 1 and Group 2 and then saw group comparison items for Group 1 versus Group 2. Students in the second version only saw the group comparison items. We found that students assigned to both versions responded similarly to the group comparison questions, indicating that the individual evaluation questions did not promote additional critical thinking. We visually represent these similarities across versions with and without the individual evaluation questions in Fig 4 as heat maps.

We ran chi-square goodness-of-fit tests on the answers between student responses on both instrument versions and there were no significant differences on the Eco-BLIC bass-mayfly scenario (Fig 4A; based on an adjusted *p*-value of 0.006) or owl-mouse questions (Fig 4B; based on an adjusted p-value of 0.004). There were only three significant differences (out of 53 items) in how students responded to questions on both versions of the PLIC (Fig 4C; based on an adjusted *p*-value of 0.0005). The items that students responded to differently (*p*<0.0005) across both versions were items where the two groups were identical in their design; namely, the equipment used (i.e., stopwatches), the variables measured (i.e., time and mass), and the number of bounces of the spring per trial (i.e., five bounces). We calculated Cramer’s C (Vc; [33]), a measure commonly applied to Chi-square goodness of fit models to understand the magnitude of significant results. We found that the effect sizes for these three items were small (Vc = 0.11, Vc = 0.10, Vc = 0.06, respectively).

The trend that students answer the Group 1 versus Group 2 comparison questions similarly, regardless of whether they responded to the individual evaluation questions, is further supported by student responses from the think-aloud interviews. For example, one participant who did not see the individual evaluation questions for the owl-mouse scenario of the Eco-BLIC independently explained that sampling mice from other fields was a strength for both the lab and field groups. They explained that for the lab group, “I think that [the mice] coming from multiple nearby fields is good…I was curious if [mouse] behavior was universal.” For the field group, they reasoned, “I also noticed it was just from a single nearby field…I thought that was good for control.” However, this individual ultimately reasoned that the field group was “more effective for sampling methods…it’s better to have them from a single field because you know they were exposed to similar environments.” Thus, even without individual evaluation questions available, students can still make individual evaluations when comparing and contrasting between groups.

We also determined that removing the individual evaluation questions decreased the duration of time students needed to complete the Eco-BLIC and PLIC. On the Eco-BLIC, the median time to completion for the version with individual evaluation and group comparison questions was approximately 30 minutes, while the version with only the group comparisons had a median time to completion of 18 minutes. On the PLIC, the median time to completion for the version with individual evaluation questions and group comparison questions was approximately 17 minutes, while the version with only the group comparisons had a median time to completion of 15 minutes.

## Discussion

To determine how to elicit critical thinking in a streamlined manner using introductory biology and physics material, we investigated (a) how students critically evaluate aspects of experimental investigations in biology and physics when they are individually evaluating one study at a time versus comparing and contrasting two and (b) whether individual evaluation questions are needed to encourage students to engage in critical thinking when comparing and contrasting.

### Students are more discerning when making comparisons

We found that students were more discerning when comparing between the two groups in the Eco-BLIC and PLIC rather than when evaluating each group individually. While students tended to independently evaluate study features of each group as strengths (Fig 2), there was greater variation in their responses to which group was more effective when directly comparing between the two groups (Fig 3). Literature evaluating the role of contrasting cases provides plausible explanations for our results. In that work, contrasting between two cases supports students in identifying deep features of the cases, compared with evaluating one case after the other [34–37]. When presented with a single example, students may deem certain study features as unimportant or irrelevant, but comparing study features side-by-side allows students to recognize the distinct features of each case [38]. We infer, therefore, that students were better able to recognize the strengths and weaknesses of the two groups in each of the assessment scenarios when evaluating the groups side by side, rather than in isolation [39, 40]. This result is somewhat surprising, however, as students could have used their knowledge of experimental designs as a contrasting case when evaluating each group. Future work, therefore, should evaluate whether experts use their vast knowledge base of experimental studies as discerning contrasts when evaluating each group individually. This work would help determine whether our results here suggest that students do not have a sufficient experiment-base to use as contrasts or if the students just do not use their experiment-base when evaluating the individual groups. Regardless, our study suggests that critical thinking assessments should ask students to compare and contrast experimental scenarios, rather than just evaluate individual cases.

### Individual evaluation questions do not influence answers to compare and contrast questions

We found that individual evaluation questions were unnecessary for eliciting or supporting students’ critical thinking on the two assessments. Students responded to the group comparison items similarly whether or not they had received the individual evaluation questions. The exception to this pattern was that students responded differently to three group comparison items on the PLIC when individual evaluation questions were provided. These three questions constituted a small portion of the PLIC and showed a small effect size. Furthermore, removing the individual evaluation questions decreased the median time for students to complete the Eco-BLIC and PLIC. It is plausible that spending more time thinking about the experimental methods while responding to the individual evaluation questions would then prepare students to be better discerners on the group comparison questions. However, the overall trend is that individual evaluation questions do not have a strong impact on how students evaluate experimental scenarios, nor do they set students up to be better critical thinkers later. This finding aligns with prior research suggesting that students tend to disregard details when they evaluate a single case, rather than comparing and contrasting multiple cases [38], further supporting our findings about the effectiveness of the group comparison questions.

### Practical implications

Individual evaluation questions were not effective for students to engage in critical thinking nor to prepare them for subsequent questions that elicit their critical thinking. Thus, researchers and instructors could make critical thinking assessments more effective and less time-consuming by encouraging comparisons between cases. Additionally, the study raises a question about whether instruction should incorporate more experimental case studies throughout their courses and assessments so that students have a richer experiment-base to use as contrasts when evaluating individual experimental scenarios. To help students discern information about experimental design, we suggest that instructors consider providing them with multiple experimental studies (i.e., cases) and asking them to compare and contrast between these studies.

### Future directions and limitations

When designing critical thinking assessments, questions should ask students to make meaningful comparisons that require them to consider the important features of the scenarios. One challenge of relying on compare-and-contrast questions in the Eco-BLIC and PLIC to elicit students’ critical thinking is ensuring that students are comparing similar yet distinct study features across experimental scenarios, and that these comparisons are meaningful [38]. For example, though sample size is different between experimental scenarios in our instruments, it is a significant feature that has implications for other aspects of the research like statistical analyses and behaviors of the animals. Therefore, one limitation of our study could be that we exclusively focused on experimental method evaluation questions (i.e., what to trust), and we are unsure if the same principles hold for other dimensions of critical thinking (i.e., what to do). Future research should explore whether questions that are not in a compare-and-contrast format also effectively elicit critical thinking, and if so, to what degree.

As our question schema in the Eco-BLIC and PLIC were designed for introductory biology and physics content, it is unknown how effective this question schema would be for upper-division biology and physics undergraduates who we would expect to have more content knowledge and prior experiences for making comparisons in their respective disciplines [18, 41]. For example, are compare-and-contrast questions still needed to elicit critical thinking among upper-division students, or would critical thinking in this population be more effectively assessed by incorporating more sophisticated data analyses in the research scenarios? Also, if students with more expert-like thinking have a richer set of experimental scenarios to inherently use as contrasts when comparing, we might expect their responses on the individual evaluation questions and group comparisons to better align. To further examine how accessible and context-specific the Eco-BLIC and PLIC are, novel scenarios could be developed that incorporate topics and concepts more commonly addressed in upper-division courses. Additionally, if instructors offer students more experience comparing and contrasting experimental scenarios in the classroom, would students be more discerning on the individual evaluation questions?

While a single consensus definition of critical thinking does not currently exist [15], continuing to explore critical thinking in other STEM disciplines beyond biology and physics may offer more insight into the context-specific nature of critical thinking [22, 23]. Future studies should investigate critical thinking patterns in other STEM disciplines (e.g., mathematics, engineering, chemistry) through designing assessments that encourage students to evaluate aspects of at least two experimental studies. As undergraduates are often enrolled in multiple courses simultaneously and thus have domain-specific knowledge in STEM, would we observe similar patterns in critical thinking across additional STEM disciplines?

Lastly, we want to emphasize that we cannot infer every aspect of critical thinking from students’ responses on the Eco-BLIC and PLIC. However, we suggest that student responses on the think-aloud interviews provide additional qualitative insight into how and why students were making comparisons in each scenario and their overall critical thinking processes.

## Conclusions

Overall, we found that comparing and contrasting two different experiments is an effective and efficient way to elicit context-specific critical thinking in introductory biology and physics undergraduates using the Eco-BLIC and the PLIC. Students are more discerning (i.e., critical) and engage more deeply with the scenarios when making comparisons between two groups. Further, students do not evaluate features of experimental studies differently when individual evaluation questions are provided or removed. These novel findings hold true across both introductory biology and physics, based on student responses on the Eco-BLIC and PLIC, respectively—though there is much more to explore regarding critical thinking processes of students across other STEM disciplines and in more advanced stages of their education. Undergraduate students in STEM need to be able to critically think for career advancement, and the Eco-BLIC and PLIC are two means of measuring students’ critical thinking in biology and physics experimental contexts via comparing and contrasting. This research offers new insight on the types of questions that elicit critical thinking, which can further be applied by educators and researchers across disciplines to teach and measure cognitive student outcomes. Specifically, we recommend instructors incorporate more compare-and-contrast questions related to experimental design in their courses to efficiently elicit undergraduates’ critical thinking.

## Supporting information

S1 Appendix Eco-BLIC bass-mayfly scenario prompt. (PDF)

S2 Appendix Eco-BLIC owl-mouse scenario prompt. (PDF)

S3 Appendix PLIC scenario prompt. (PDF)
