# Supplementary material for: What influences students’ abilities to critically evaluate scientific investigations?
Source: PLoS One. 2022 Aug 30;17(8):e0273337. doi: 10.1371/journal.pone.0273337 (PMC9426932; doi:10.1371/journal.pone.0273337)

S1 Appendix. Eco-BLIC Bass-Mayfly Scenario Prompt.

Two groups of biologists are studying smallmouth bass (*Micropterus dolomieu*) and combmouthed minnow mayflies (*Ameletus cryptostimulus*; hereafter referred to as mayflies). Smallmouth bass eat young mayflies, which live at the water surface. Mayflies do not grow bigger than what smallmouth bass can eat. Both groups of biologists want to know whether smallmouth bass selectively feed on larger or smaller mayflies.

Bass/Mayfly FIELD scenario: One group surveys 30 ponds in the field (15 with smallmouth bass and 15 without smallmouth bass); *see image below for visual*. The researchers measure mean length of 10 mayflies from each pond and find the pattern displayed in the following figure:

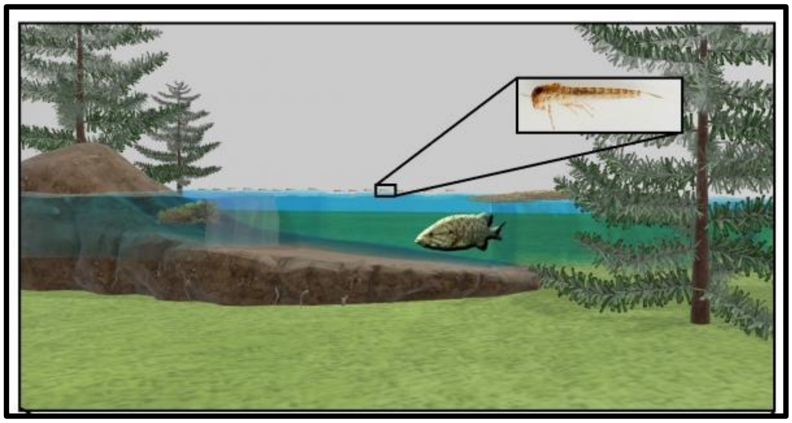

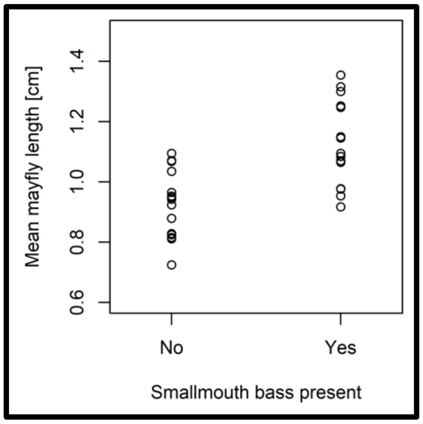

Bass/Mayfly LAB scenario: A second group collects smallmouth bass and mayflies from one pond in the field and brings these organisms back to the lab. The researchers set up 10 tanks with 100 mayflies and placed one bass per tank for 24 hours; *see image below for visual.* They took a random sample of 20 mayflies from each tank before and after this period and find the pattern displayed in the following figure:

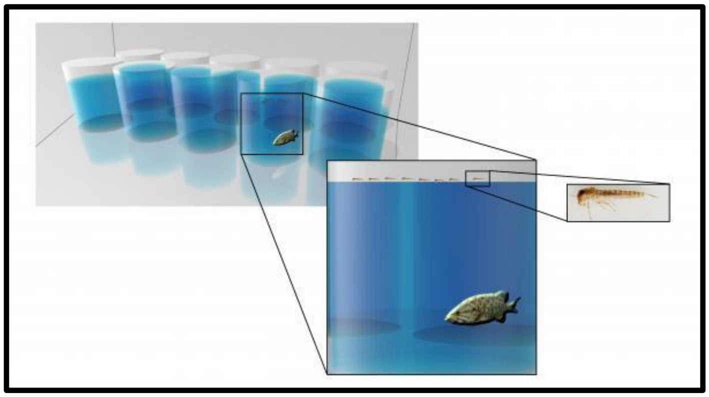

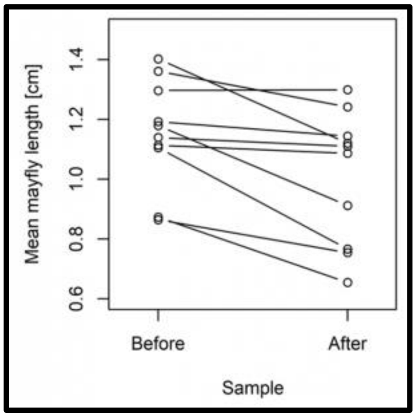

Supplement: S1 Appendix — (PDF) [file pone.0273337.s001.pdf]
