# Supplementary material for: What influences students’ abilities to critically evaluate scientific investigations?
Source: PLoS One. 2022 Aug 30;17(8):e0273337. doi: 10.1371/journal.pone.0273337 (PMC9426932; doi:10.1371/journal.pone.0273337)

S2 Appendix. Eco-BLIC Owl-Mouse Scenario Prompt.

Two groups of biologists are studying the feeding behavior of the house mouse (*Mus musculus*, hereafter referred to as mouse/mice) in the presence or absence of one of its natural predators, the great-horned owl (*Bubo virginianus*). Mice have a strong sense of smell and hearing and can be social or solitary, depending on living conditions. Mice commonly feed on seeds. Both species are nocturnal and generally feed at night. The two groups of biologists want to know how the presence of a great-horned owl influences the amount of time that mice spend feeding.

Owl/Mouse LAB scenario: One group conducts their study in two rooms in the lab. In both rooms, there is a single cage, each with 5 mice. In each cage, there is a large bowl of seeds and nest box for sleeping, and a camera is attached to the cage to track mice moving in and out of their next box. However, while there is no perceived risk of predation in room one, the researchers play great-horned owl calls in room two every 15 minutes for 30 seconds. *See image below for visual*. The researchers measure average time at food bowl in each room and find the pattern displayed in the following figure:

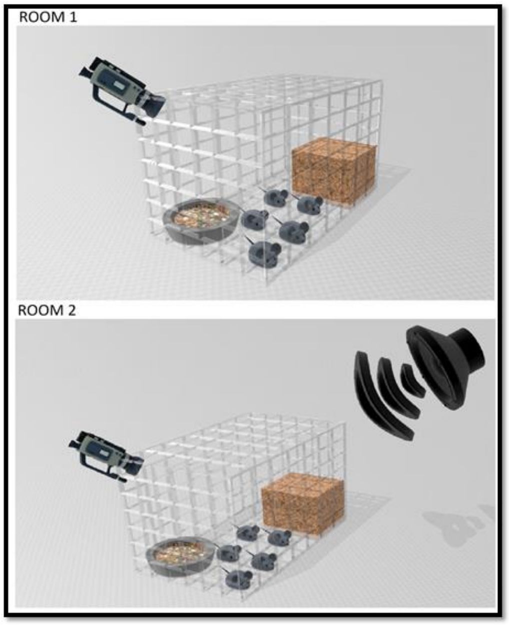

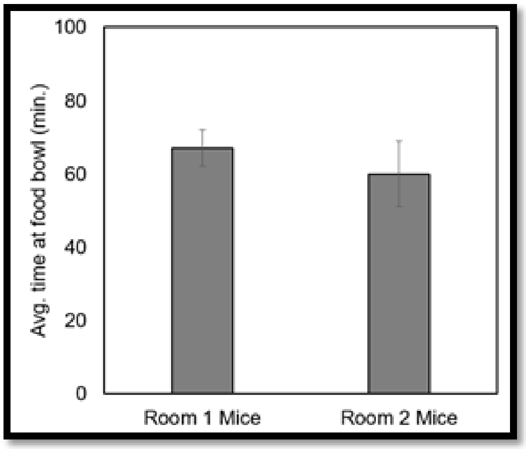

Owl/Mouse FIELD scenario: The second group conducted their study in an outdoor enclosure. They set up 15 cages, each with a single mouse. In each cage, there is a large bowl of seeds and nest box for sleeping, and a camera is attached to the cage to track mice moving in and out of their next box. One night one, there is no perceived risk of predation; on night two, the researchers allow a great-horned owl to fly freely around the outdoor enclosure. *See image below for visual*. The researchers measure total time at food bowl on each night and find the pattern displayed in the following figure:

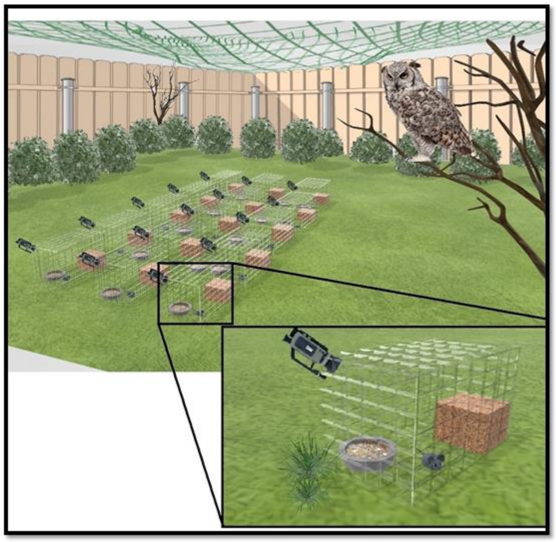

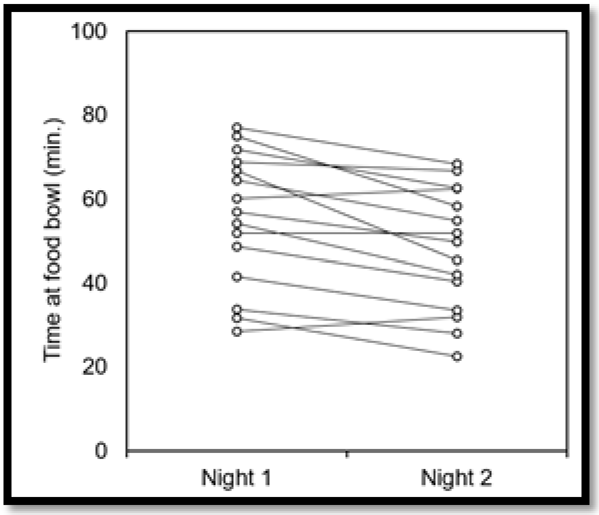

Supplement: S2 Appendix — (PDF) [file pone.0273337.s002.pdf]
