# Supplementary material for: What influences students’ abilities to critically evaluate scientific investigations?
Source: PLoS One. 2022 Aug 30;17(8):e0273337. doi: 10.1371/journal.pone.0273337 (PMC9426932; doi:10.1371/journal.pone.0273337)
Supplement: S3 Appendix — (PDF) [file pone.0273337.s003.pdf]

S3 Appendix. PLIC Scenario Prompt.

Introduction:

In the following questions, you will explore two groups of physicists conducting an experiment about a mass on a spring.

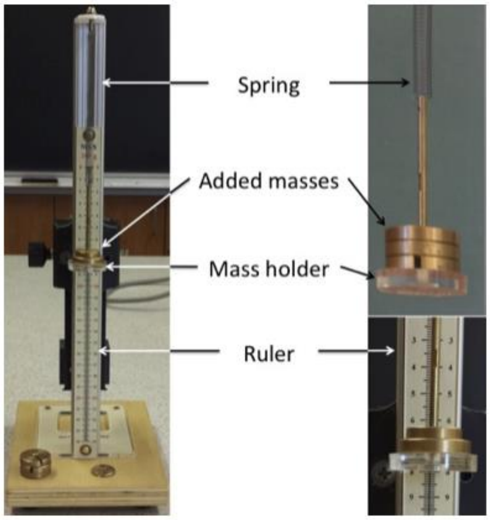

If a mass, m, on a spring with spring constant k is pulled down from the spring's equilibrium position, it oscillates (or bounces). The period, T, of this bouncing is modeled as

$$T = 2\pi\sqrt{\frac{m}{k}}.$$

Two groups of physics are designing and carrying out experiments to test how well a mass on a spring obeys this model. The pictures on the right show their set ups.

The two groups are able to come up with their own experimental designs and have the following equipment available to them:

- a digital stopwatch (0.01 s increments),
- a spring with unknown spring constant, k,
- a mass holder (10 g) attached to the spring,
- a set of masses,
- and a ruler attached to the spring holder (1 mm increments).

The following questions will ask you about the two groups' experiments. You will not be able to revisit previous questions as you move through the questions.

Group 1 Scenario:

Below are the notes Group 1 made in their lab book. You will then be asked about their data and methods. Click here to see the introduction again.

Method To test the model ($T = 2\pi\sqrt{\frac{m}{k}}$), we found the period, T, for two different masses, then calculated and compared the two k-values (the values should be the same because it's the same spring).

First, we put 20 g on the holder (holder is 10 g, so total is 30 g)

Used the stopwatch to measure the time for 5 bounces (periods) and divided the time by 5 to get the period, T. Repeated 10 times.

Then repeated with 40 g (instead of 20 g) on the holder (total of 50 g). Table below shows data.

Total mass: 30 g

Time for 5 oscillations (s)	Period (s)
2.78	0.556
2.68	0.536
2.76	0.552
2.70	0.540
2.67	0.534
2.82	0.564
2.66	0.532
2.72	0.544
2.56	0.512
2.57	0.514

T(average) = 0.5384 ± 0.00531 s

$\Rightarrow k = \frac{4\pi^2 m}{T^2} = 4.086 \pm 0.0807$ N/m

Total mass: 50 g

Time for 5 oscillations (s)	Period (s)
3.39	0.678
3.35	0.670
3.40	0.680
3.42	0.684
3.40	0.680
3.45	0.690
3.31	0.662
3.55	0.710
3.60	0.720
3.46	0.692

T(average) = 0.6866 ± 0.00553 s

$\Rightarrow k = \frac{4\pi^2 m}{T^2} = 4.187 \pm 0.0675$ N/m

Group 2 Scenario:

Below are the notes Group 2 made in their lab book. You will then be asked about their data and methods. Click here to see the introduction again and here to see Group 1's lab book again.

Method

To test the model ($T = 2\pi\sqrt{\frac{m}{k}}$), we used the stopwatch to measure two trials of the time for 5 bounces for 10 different masses (0.010 kg to 0.1 kg).

Timing uncertainty is about 0.1 s.

Based on the model, plotting our data as period-squared (T^2) versus mass should give a best-fit line with an intercept through the origin (because $T^2 = 4\pi^2\frac{m}{k}$).

If we fix the y-intercept at 0, the best-fit line slope should be $\frac{4\pi^2}{k}$

Best-fit line info:

slope $= 9.7393 \pm 0.1683$ m/N $= \frac{4\pi^2}{k}$

$\Rightarrow$ k$= \frac{4\pi^2}{slope} = 4.0535 \pm 0.07005$ N/m

The bottom plot shows how far each data point is from the line (difference between measured value and value at the best-fit line)

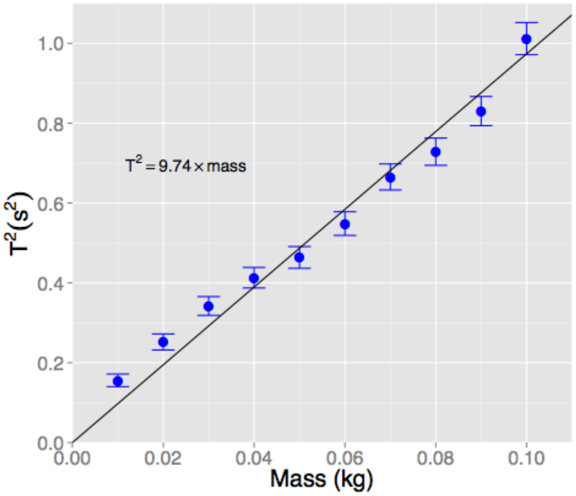

(a) Data and fit line

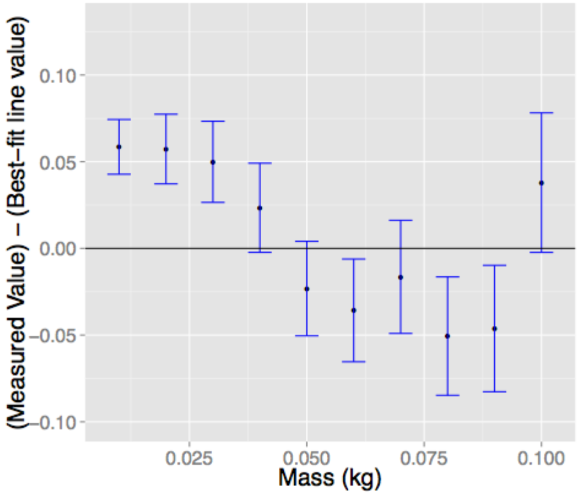

(b) Difference between measured values and best-fit line values

Below are additional notes Group 2 made in their lab book. You will then be asked about their data and methods. Click here to see the intro again, here to see Group 1's lab book, and here to see Group 2's original lab book notes.

What happens if we add a non-zero intercept to the fit (new graphs below)?

slope = 8.5314 $\pm$ 0.29259 m/N $\Rightarrow$ k= $\frac{4\pi^2}{slope}$ = 4.6274 $\pm$ 0.1587 N/m

intercept = 0.07051 $\pm$ 0.01397 s^2

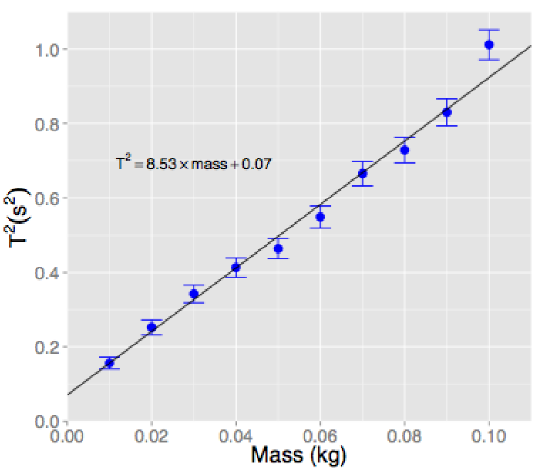

(a) Data and fit line (intercept not set to 0)

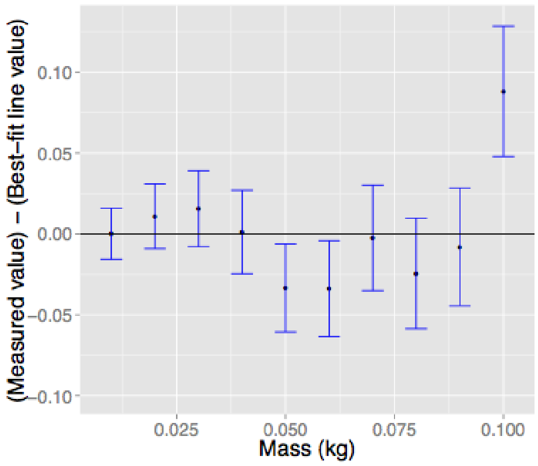

(b) Differences between measured values and best-fit line values
